# Comparison Between the NIST and the KEBS for the Determination of Air Kerma Calibration Coefficients for Narrow X-Ray Spectra and ^137^Cs Gamma-Ray Beams

**DOI:** 10.6028/jres.115.002

**Published:** 2010-02-01

**Authors:** Michelle O’Brien, Ronaldo Minniti, Stanslaus Alwyn Masinza

**Affiliations:** National Institute of Standards and Technology (NIST), Gaithersburg, MD 20899 USA; Kenya Bureau of Standards (KEBS), Nairobi, Kenya

**Keywords:** air kerma, free-air ionization chamber, gamma-ray, cesium 137, primary standard, reference radiation qualities, x-ray calibration

## Abstract

Air kerma calibration coefficients for a reference class ionization chamber from narrow x-ray spectra and cesium 137 gamma-ray beams were compared between the National Institute of Standards and Technology (NIST) and the Kenya Bureau of Standards (KEBS). A NIST reference-class transfer ionization chamber was calibrated by each laboratory in terms of the quantity air kerma in four x-ray reference radiation beams of energies between 80 kV and 150 kV and in a cesium 137 gamma-ray beam. The reference radiation qualities used for this comparison are described in detail in the ISO 4037 publication.[1]

The comparison began in September 2008 and was completed in March 2009. The results reveal the degree to which the participating calibration facility can demonstrate proficiency in transferring air kerma calibrations under the conditions of the said facility at the time of the measurements. The comparison of the calibration coefficients is based on the average ratios of calibration coefficients.

## 1. Introduction

The objective of this proposal was to compare the air kerma calibration coefficients of a reference class chamber determined in reference beams of x rays and gamma rays. The comparison was performed between the National Institute of Standards (NIST) and the Kenya Bureau of Standards (KEBS). A NIST reference-class transfer ionization chamber was calibrated by each laboratory in terms of the quantity air kerma in four ISO narrow x-ray spectra beams with energies between 80 kV and 150 kV and in a ^137^Cs gamma-ray beam. The reference radiation qualities used for this comparison are described in detail in the ISO 4037 publication [1].

## 2. Procedure

### 2.1 Object of Comparison

The object was to compare the value of the air kerma calibration coefficient of the chamber determined at the KEBS with the value obtained at the NIST. The transfer chamber used during the comparison was an Exradin^1^ chamber model A5, SN XY051605.

### 2.2 Transfer Chamber

The chamber used is spherical and made of C552 air equivalent plastic and has a nominal volume of 100 cm^3^. The air kerma calibration coefficient *N_K_* was determined by evaluating the ratio *N_K_* = *K*_air_/*I*_corr_, where *K*_air_ is the reference air kerma rate (expressed in units of Gy/s) and *I*_corr_ is the measured ionization current (expressed in units of C/s) corrected for influence quantities. A bias voltage of 400 volts was applied to the chamber. This is the maximum voltage allowed by the electrometer owned by the KEBS. Table 1 summarizes the chamber specifications.

### 2.3 Reference Radiation Qualities

The measurements made at the NIST were performed in the x-ray and gamma-ray beam calibration facilities. The air kerma rates at both of these facilities are well established and are determined using the NIST primary standard instruments. Details on the determination of the air kerma rates using the primary standard instruments and the NIST calibration service can be found in Refs. [2–5].

For the x-ray measurements, the air kerma rates were determined directly using the Wyckoff-Attix free-air chamber. This primary standard for x rays is located in the NIST 300 kV calibration facility. The x-ray source used at the time of the comparison was a 320 kV x-ray generator with a metal ceramic x-ray tube, both supplied by Seifert-Pantak.^1^ The x-ray generator is a high-frequency, highly stabilized voltage source. The tungsten anode x-ray tube, model MXR-321, has a beryllium window of thickness 3 mm and a focal spot 8.0 mm in diameter. The materials used for the filtration and for the measurement of Half Value Layer (HVL) were at least 99.99 % pure with thicknesses known with an uncertainty of 0.01 mm. The reference radiation qualities used for the comparison are described in ISO 4037 as the narrow spectra [1]. The NIST and the KEBS HVL’s are listed in Table 2.

The gamma-ray measurements were made in the NIST ^137^Cs horizontal beam gamma-ray calibration facility. The air kerma rates in this facility were obtained by applying a decay correction to the reference air kerma rate. The reference air kerma rates were determined using a suite of six graphite wall cavity ionization chambers. The beam is collimated providing a circular beam size of 15 cm radius at a source-to-detector distance of 195 cm. The beam is uniform over the area across the chamber used in this work. Various types of reference class ionization chambers are used routinely to track the reference air kerma rate value.

#### 2.4.1 Reference Conditions at the NIST

At the NIST each x-ray calibration was made by alternating between the transfer chambers and the standard free-air chamber, through the translation of the chambers to one beam center line. Beam alignment to the axis is accepted to be 0.1 mm and reproducible to better than 0.01 mm, as observed by an alignment telescope. The reference plane for all measurements at the NIST was positioned at 1000 mm from the x-ray radiation source and 1950 mm from the ^137^Cs source. The beam diameter in the reference plane was at least 90 mm for the x-ray measurements and at least 150 mm for the gamma-ray measurements. The exact beam geometry was not prescribed in the protocol so that the KEBS could use the appropriate geometry depending on the field size and field uniformities available and the conditions that best represents routine calibrations at their facility. The influence of field size was not investigated for this comparison. The geometry and conditions of the air kerma measurement were kept consistent at NIST for all measurements. Table 3 describes the radiation reference field sizes used for the comparison.

At the NIST the chamber background current was measured before and after the chamber was exposed to the source of radiation. The average of the background currents was determined and subtracted from the measured ionization current produced in the chamber when exposed to the source of radiation. Typical background currents were of the order of 0.01 % of the ionization current. The integration times used at the NIST were 120 seconds for the x-ray measurements and 60 seconds for the gamma-ray measurements. The measured ionization currents were normalized to 295.15 K and 101 325 Pa. The humidity was monitored and recorded and was typically in a range of 30 %, however the currents were not corrected for humidity. Only the negative polarity measurements were used for the comparison results, resulting in a negative charge.collected on the electrometers. No correction was applied to the calibration coefficient to account for polarity effect since at both facilities the same polarity was used. The NIST performed measurements at both polarities to verify the influence of the polarity effect. The value of the calibration coefficients obtained with both polarities agreed within less than 0.10 % for all reference beams used. This implies that the polarity effect is negligible. No corrections were applied to the calibration coefficients to account for electronic recombination effects. However an estimate of the recombination correction was obtained using the two-voltage method. For this, the ratio of the ionization currents measured at the full voltage of 400 V and half voltage of 200 V was obtained.

The KEBS was requested to provide the calibration coefficients for the transfer chambers in terms of air kerma per charge in units of Gy/C and refer to standard conditions of air temperature at 295.15 K, pressure at 1013.25 hPa and 50 % relative humidity. The KEBS was requested to use the same polarity for the voltage bias of the Exradin chamber.

#### 2.4.2 Reference Conditions at the KEBS

X-ray calibrations for each beam quality were made by alternating between the transfer chambers and the standard free-air chamber, through the translation of the chambers to the central beam axis indicated by the laser beam. The details of the KEBS standard can be found in Ref. [6]. The x-ray apparatus at KEBS consists of several elements placed between the x-ray tube and the detection distance. Starting at the x-ray source these elements include a diaphragm/aperture wheel holder at 200 mm from the source, a filter wheel holder at 220 mm, a permanent tube inherent filter holder at 230 mm, a monitor chamber at 240 mm, and a HVL filter holder. The HVL diaphragm holder is only used for HVL measurements and is placed at 630 mm from the tube. The diaphragm holder is remotely adjustable from the control unit. The filters can be selected from the control unit. The comparison ionization chamber was placed at the measuring distance of 2000 mm from the source. Details of the KEBS laboratory setup can be found in Ref. [7].

The gamma-ray measurements were made in the KEBS ^137^Cs gamma-ray calibration laboratory. The air kerma rates in this facility are obtained by applying a decay correction to the reference air kerma rates that were established on July 2007, at the time the facility was commissioned. These daily air kerma rate values are tracked using a laboratory developed spreadsheet and confirmed through routine measurements using ionization chambers models LS01 and LS10 as part of the quality control of the facility. It is important to mention that the air kerma standards at KEBS are traceable indirectly to the Bureau International des Poids et Measures (BIPM) standards through the International Atomic Energy Agency (IAEA). The reference air kerma rates at KEBS were determined using the KEBS secondary standard chamber model W-32002, SN 247 calibrated at the International Atomic Energy Agency (IAEA). The value of the most recent ^137^Cs calibration coefficient of the KEBS standard chamber obtained from IAEA is 2.52 × 10^4^ Gy/C. This value includes a correction introduced by the BIPM in the year 2009 that applies to all calibration facilities that are directly and indirectly traceable to the BIPM, including KEBS. Furthermore, all results presented in this work and obtained at KEBS, include all corrections introduced by the BIPM up to the date of this publication.

The KEBS gamma beam geometry uses a collimated circular beam with a radius of approximately 340 mm at a source-to-detector distance of 1950 mm. The beam is uniform over the area across the chamber. At a distance of 1000 mm, the variation of the beam intensity across a circular area of 2850 mm does not exceed 5 % when the largest collimator is used.

The NIST chamber was connected to the PTW electrometer. Prior to performing the measurements, a voltage was applied to the chamber overnight to allow sufficient time for the chamber to stabilize. Background currents were measured before and after the chamber was exposed to the source of radiation. The average of the background currents was determined and subtracted from the measured ionization current produced in the chamber when exposed to the source of radiation. Typical background currents were of the order of 0.02 % of the ionization current. The integration time used at the KEBS was 60 seconds for both the x-ray and the gamma-ray measurements. The measured ionization currents were normalized to 295.15 K and 101 325 Pa. The humidity was monitored and recorded and was typically in a range of 40 %, however the currents were not corrected for humidity. The calibration coefficients for the transfer chamber were given in terms of air kerma per charge in units of Gy/C and corrected to standard conditions.

### 2.5 Course of Comparison

The NIST shipped the chamber to the KEBS in September 2008 with a tentative scheduled return to the NIST of November 2008. However, due to unexpected delays at the KEBS resulting from equipment failure the measurements were completed in March 2009. After completion of the calibrations at the KEBS facility and the return of the chamber to the NIST, constancy checks were performed in late March and early April of 2009. The values of the calibration coefficients obtained at the NIST before and after the shipment of the chamber to the KEBS agreed as expected, indicating that there were no alterations or damage to the chamber during the full period of the comparison.

## 3. Uncertainties

The uncertainties associated with the NIST primary x-ray standard are listed in Table 4. Following standard guidelines for the expression of uncertainity published elsewhere [8,9], the uncertainties listed in the tables in this work are classified in Type A and Type B according to the method used to evaluate them. Type A uncertainties, denoted as *u_i_*_A_, are determined from statistical analysis while Type B uncertainties, denoted as *u_i_*_B_, are determined by other means such as scientific judgment. As shown in the table, the relative fractional combined uncertainty of the air kerma rate, ${\dot{K}}_{\text{Standard}}$, from the x-ray beam is 0.0030. Similarly, the relative combined uncertainty for the air kerma rate, ${\dot{K}}_{\text{Standard}}$,from the ^137^Cs gamma ray beam is 0.0029 (as listed in Table 7 ahead). This results in relative combined uncertainties of the air kerma rates of 0.30 % and 0.29 % for x rays and gamma rays respectively when expressed as a percentage. Table 5 and Table 6 show the uncertainty analysis associated with the calibration of the transfer chamber at the NIST and the KEBS using x rays. The uncertainty of the calibration is given by the uncertainty of the calibration coefficient *N_K_*. Similarly Table 7 and 8 show the uncertainty analysis associated with the calibration of the chamber at both facilities in the ^137^Cs beam. Note that in Tables 7 and 8, the relative uncertainties of the chamber current corrected for influence quantities and the air kerma rate value provided by the standard, are combined, resulting in the relative standard uncertainty of the calibration coefficient *N_K_*. Finally, Table 9 lists the relative combined uncertainty of the comparison ratio, KEBS/NIST, obtained both with x-rays and gamma-rays. The uncertainty of the calibration coefficients *N_K_* for the x-ray portion of the comparison listed in Table 9 are grouped in Type A and Type B evaluations, whereas the uncertainties for the gamma ray calibration coefficients are expressed directly as the relative combined uncertainties.

## 4. Results and Discussion

The mean value of the calibration coefficients *N_K_* measured at the NIST and the KEBS are shown in Table 10. The average air kerma rates used at both facilities are listed in Table 11. The comparison results are listed in Table 12 as the ratio of the mean calibration coefficients, *N_K_*_,KEBS_ / *N_K_*_,NIST_ for each reference radiation with an associated comparison uncertainty listed in Table 9. The mean ratio of the comparison ratios for the x rays is 0.984. The corresponding standard deviation of the four comparison ratios is 0.0083. Acceptable agreement is also achieved for the gamma-ray comparison with a resulting ratio of 1.006 with a standard uncertainty of the ratio of 0.0134.

The differences observed in the values of the calibration coefficients, resulting in agreement of approximately 1.6 % for x rays and 0.6 % for gamma rays, can be due to the variations in the field sizes and source to detector distances used at both facilities. Although the beam uniformity and scatter characteristics at NIST meet the conditions as described in the ISO 4037 [1], measurements at the NIST, using the Exradin A5 chamber at various distances and field sizes, result in differences of the calibration coefficient of up to 1 %. Since this comparison was not designed to compare beam geometry, the uncertainty due to differences in field sizes and scatter conditions was not directly addressed.

The polarity effect and the electronic recombination effect were excluded as possible influences to the comparison results. The differences due to the polarity of the comparison chamber bias voltage were measured to have a negligible effect for the specific chamber volume and the rates used for the comparison. The air kerma rates used at both laboratories were similar, resulting in probable similar electronic recombination effects. The estimate of the recombination effect obtained using the two-voltage method resulted in negligible influences. The uncertainty analysis did not include these negligible components.

## Figures and Tables

**Table 1:** Description of the chamber

Type	Serial Number	Sensitive volume (nominal	Outside Diameter (nominal)	Diameter of inner electrode	Chamber Voltage
Exradin A5	XY051605	100 cm^3^	6 cm	6 mm	400 V

**Table 2:** Characteristics of reference radiation qualities

Reference Radiation	Generating tube potential (kV)	NIST	KEBS
		Half-value layer (mm Cu)	
NS80	80	0.59	0.56
NS100	100	1.14	1.07
NS120	120	1.76	1.67
NS150	150	2.41	2.34
Cs-137	Not Applicable	10.8	10.9

**Table 3:** Beam geometry used at both facilities

Geometry	NIST	KEBS	NIST	KEBS
	X-ray spectra		^137^Cs	
Calibration distance (mm)	1000	2000	1950	1950
Beam diameter (mm)	90	853	150	682.5

**Table 4:** Uncertainties associated with the NIST standard for the x-ray spectra

Relative standard uncertainty	NIST standard	
	*u_i_*_A_	*u_i_*_B_
Ionization current	0.0008	0.001
Volume	0.0004	0.0001
Positioning		0.0001
Correction factors (excl. *k*_h_)	0.0012	0.0024
Humidity *k*_h_		0.0003
Physical constants		0.0015

${\dot{K}}_{\text{Standard}}$	0.0015	0.003
	0.003	

**Table 5:** Uncertainties associated with the NIST calibration of the A5 Exradin transfer chamber in the x-ray beam

Relative standard uncertainty	NIST standard	
	*u_i_*_A_	*u_i_*_B_
${\dot{K}}_{\text{Standard}}$	0.0015	0.0030
Ionization current	0.00083	0.0010
Positioning	0.0001	
Humidity		0.0003

	0.0015	0.0032
*N_K_*_,NIST_	0.00354	

**Table 6:** Uncertainties associated with the KEBS calibration of the A5 Exradin transfer chamber in the x-ray beam

	KEBS	
Relative standard uncertainty	*u_i_*_A_	*u_i_*_B_
	KEBS Reference chamber	
*N_K_*_,reference_	0.00200	
Long term stability	0.00052	
Positioning		0.00290
Ionization current	0.00210	
Temperature and pressure	0.00003	
	Exradin chamber at KEBS	
Positioning		0.00290
Temperature and pressure	0.00070	
Ionization current	0.00667	

*N_K,KEBS_*	0.00732	0.00410
	0.00839	

**Table 7:** Uncertainties associated with the NIST calibration of the A5 Exradin transfer chamber in the ^137^Cs gamma-ray beam

Relative standard uncertainty	NIST	
	*u_i_*_A_	*u_i_*_B_
Charge	0.0010	0.0010
Time		0.0005
Air density correction (temperature and pressure)		0.0003
Distance		0.0002
*k*_sat_, loss of ionization due to recombination	0.0001	0.0005
Probe orientation		0.0001
Humidity		0.0006

	0.0010	0.0014
Chamber current corrected for all influence quantities		
	0.0017	
${\dot{K}}_{\text{Standard}}$	0.0029	
*N_K_*_,NIST_	0.0034	

**Table 8:** Uncertainties associated with the KEBS calibration of the A5 Exradin transfer chamber in the ^137^Cs gamma-ray beam

Relative standard uncertainty	KEBS	
	*u_i_*_A_	*u_i_*_B_
Stability of reference value *K*_air_ at 2 m	0.0024	0.0000
Temperature and pressure	0.0003	0.0015
Leakage current	0.0000	0.0100
	0.0024	0.0.0101

Chamber current corrected for all influence quantities		
	0.0104	
${\dot{K}}_{\text{Standard}}$	0.0078	
*N_K_*,NIST	0.0130	

**Table 9:** Uncertainties associated with the comparison results

Relative standard uncertainty	X-rays		Cs-137
	*u_i_*_A_	*u_i_*_B_	*u_i_*
*N_K_*_,KEBS_	0.0073	0.0041	0.0130
*N_K_*_,NIST_	0.0015	0.0032	0.0034
*N_K_*_,KEBS_ / *N_K_*_,NIST_	0.0075	0.0052	0.0134

${\dot{K}}_{\text{KEBS}}/{\dot{K}}_{\text{NIST}}$	0.0091		0.0134

**Table 10:** Mean values of the calibration coefficients measured at the NIST and the KEBS using the Exradin A5

Reference radiation	Calibration coefficients 10^5^ GyC^−1^	
	NIST Mean *N_K_*	KEBS Mean *N_K_*
NS100	2.969	2.886
NS80	2.929	2.898
NS100	2.969	2.886
NS120	2.999	2.961
NS150	2.991	2.955
Cs-137	3.047	3.065

**Table 11:** Average air kerma rates used at both facilities

Reference radiation	Average air kerma rate 10^−6^ Gys^−1^	
Beam	NIST	KEBS
pNS80	6.61	3.74
NS100	4.08	2.87
NS120	4.50	1.59
NS150	3.53	12.9
Cs-137	26.0	3.31

**Table 12:** Results of comparison

Reference radiation	Mean *N_K_*_,KEBS_ / *N_K_*_,NIST_
NS80	0.990
NS100	0.972
NS120	0.987
NS150	0.988
Cs-137	1.006

## Footnotes

1 Certain commercial equipment, instruments, or materials are identified in this paper to foster understanding. Such identification does not imply recommendation or endorsement, nor does it imply that the equipment identified are necessarily the best available for the purpose.
